# Role of *BmDredd* during Apoptosis of Silk Gland in Silkworm, *Bombyx mori*

**DOI:** 10.1371/journal.pone.0169404

**Published:** 2017-01-09

**Authors:** Rui-ting Chen, Peng Jiao, Zhen Liu, Yan Lu, Hu-hu Xin, Deng-pan Zhang, Yun-gen Miao

**Affiliations:** Institute of Sericulture and Apiculture, College of Animal Sciences, Zhejiang University, Hangzhou, People's Republic of China; Institute of Plant Physiology and Ecology Shanghai Institutes for Biological Sciences, CHINA

## Abstract

Silk glands (SGs) undergo massive apoptosis driven degeneration during the larval-pupal transformation. To better understand this event on molecular level, we investigated the expression of apoptosis-related genes across the developmental transition period that spans day 4 in the fifth instar *Bombyx mori* larvae to day 2 pupae. Increases in the expression of *BmDredd* (an initiator caspase homolog) closely followed the highest *BmEcR* expression and resembled the expression trend of *BmIcE*. Simultaneously, we found that *BmDredd* expression was significantly higher in SG compared to other tissues at 18 h post-spinning, but reduced following injection of the apoptosis inhibitor (Z-DEVD-fmk). Furthermore, *BmDredd* expression correlated with changes of caspase3-like activities in SG and RNAi-mediated knockdown of *BmDredd* delayed SG apoptosis. Moreover, caspase3-like activity was increased in SG by overexpression of *BmDredd*. Taken together, the results suggest that *BmDredd* plays a critical role in SG apoptosis.

## Introduction

Programmed cell death (PCD) is a vital biological event accompanied by characteristic events including chromatin condensation, DNA fragmentation, vacuolization, and apoptotic body formation [1]. Competence of PCD is achieved by the activation of the cysteine protease (caspase) cascade. Therefore, caspases are indispensable essential during cell death [2, 3]. In insects, larval specific tissues are eliminated through PCD [4], which is triggered by endocrine hormones that lead to activation or repression of a succession of intracellular factors [5–7].

Silkworm *Bombyx mori* is an important economic insect and model organism which has been used effectively in various biological researches [8]. The economic importance of silkworms is dependent on the functionality of the silk gland (SG), a specialized organ that synthesizes silk proteins in the silkworm. The SG undergoes rapid development during the fifth instar larvae with the genomic DNA content in SG cells increasing 200–400000 times through the process of endomitosis [9, 10]. Consequently, the SG becomes the largest organ in silkworm abdominal cavity until the wandering stage. Then the SG degenerates rapidly after the spinning stage in response to endocrine hormones (including juvenile hormone and ecdysone) [4, 11, 12]. This process includes 5 characteristics (nuclear condensation, DNA fragmentation, nuclear fragmentation, cells chrinkage and apoptotic body formation) and involves in PCD [7]. Among which, ecdysone (20E, 20-Hydroxyecdysone) plays a leading role by acting through binding to ecdysone receptor (EcR) / ultraspiracle (USP) complex. This 20E-induced PCD is similar with the mechanism found in *Drosophila*, where 20E/EcR/USP complex regulates primary-response genes including a number of transcription factors such as E74, Board complex (BR-C) and E93, which directly regulate secondary-response target genes to transmit and amplify the hormonal signal causing apoptosis [13]. In addition, 20E-induced SG degeneration (mainly nuclear condensation and DNA fragmentation) could be inhibited following addition of a caspase3 inhibitor to the culture medium [7]. Consequently, it is clear that 20E induced cell death involves different temporal stages in both *Drosophila* and silkworms [7, 13, 14], and the regulation of diverse genes [15–17]. Apart from this, we could know little details about the molecular mechanism of SG apoptosis.

Apoptosis proceeds via an intrinsic pathway that involves mitochondrial cytochrome C (Cyt C) release and an extrinsic pathway commonly [18]. Many studies have proven that the Cyt C might play little role in regulating apoptosis in *Drosophila*. In contrast to that reported in *Drosophila*, Cyt C release importantly participates in silkworm apoptosis, suggesting that silkworms may be better model systems for apoptosis research [17, 19, 20]. So far, several studies have elucidated that some homologous genes in silkworms also function similarly as in mammals. *Bm*Ice-2, *Bm*p53 [20] and *Bm*Dronc [21] have been reported to enhance the rate of apoptosis in *Bm*N cells, while *Bm*IAP [22] and *Bm*Buffy [23] function in inhibition of apoptosis. *Bombyx mori* death related ced-3/Nedd2-like protein (*Bm*Dredd) has relatively high homology with the mammalian initiator caspases which include an N-terminal death effector domain (DED) or caspase recruitment domain (CARD) and a C-terminal CASc domain. The difference is that *Bm*Dredd comprises a long prodomain which replaces the mammalian N-terminal DED or CARD. Initiator caspases are gathered in a close distance through DED or CARD and finally activated by autoproteolysis in its CASc domain [24]. Recently, some evidence has showed that *Bm*Dredd functions importantly in apoptosis. Its expression level was elevated in actinmycin D induced *Bm*E cells [17] and in actinmycin D or ultraviolet induced *Bm*N cells [25]. In addition, overexpression of *Bm*Dredd caused the increase of caspase3/8-like protease activity in *Bm*N-SWU1 cells [26]. However, a more advanced understanding of its role during SG degeneration has eluded researchers due to the instability of animal individual experiments. In this study, we addressed this deficiency by examining the role of *BmDredd* in apoptosis of the SG.

## Materials and Methods

### Animals

Silkworm larvae (P50 strain) were reared on fresh mulberry leaves. Silkworms were maintained at 25 ± 1°C, 70–85% humidity, and a 14 h light/10 h dark photoperiod.

### RNA extraction & cDNA synthesis

Tissues (SG, gut, fat, gonad and epidermis) were dissected in PBS buffer and ground under liquid nitrogen. Total RNA was isolated using RNAiso Plus according to the manufacturer’s instructions (TaKaRa, Japan) and measured on a Bio Spec-nano (Shimadzu Biotech, Japan). For each sample, 500 ng of total RNA was used to synthesize the first cDNA strand using a Prime Script RT Master Mix (TaKaRa) according to the manufacturer’s instructions.

### qRT-PCR

The *BmDredd* nucleotide sequences (accession number: NM_001114865) were searched against the NCBI (http://www.ncbi.nlm.nih.gov/pubmed/) database and primers were designed by primer 5.0 (Premier Biosoft International, Palo Alto, CA, USA) and synthesized by Sangon Biotech, Shanghai, China (Table A in S1 File). qRT-PCR was performed in a total volume of 20 μl on an ABI7300 System (Applied Biosystems, Foster City, CA, USA) and SYBR^®^Premix Ex Taq^TM^ (TaKaRa) using cycling parameters consisting of an initial denaturation at 95°C/30 sec followed by 40 cycles of 95°C/5 sec and 60°C/31 sec. A melt curve analysis was used to confirm the absence of spurious products. Ct values were employed to calculate the relative expression levels [27]. Transcription levels of the target genes were normalized with *Actin3* transcription levels from the same samples. All of the experiments were performed in triplicate.

### Caspase inhibitor and ecdysone treatment

The caspase inhibitor, Z-DEVD-fmk (Selleck Chemicals, Houston, TX, USA), was diluted in PBS according to the manufacturer instructions and then injected a corresponding quantity into day 7 of the fifth instar (L5D7) silkworms based on its weight (22.4 μg per gram) and SGs were dissected at 18 h-post the spinning (sp18h). Similarly, ecdysone (Huzhou Silkworm Pharmaceutical Factory, China) was diluted with ddH_2_O and sprayed evenly onto mulberry leaves according to the instruction (~5 μg each silkworm). Feed the leaves to experimental silkworms.

### Protein extraction and caspase3 like activity assay

Dissected SGs were ground under liquid nitrogen and homogenized in100 μl tissue lysis buffer (Beyotime, Shanghai, China) per 5–10 mg tissue. Lysates were then placed on an ice bath for 5 min and centrifuged at 16000–20000 ×*g* at 4°C for 15 min. The supernatant was transferred to ice cooled 1.5 ml tubes for assaying the caspase3 activity, and the total protein concentration was determined using a Bradford Protein Assay Kit (TaKaRa). Samples were maintained at -80°C.

According to the instruction of Caspase3 Activity Assay Kit (Beyotime), 50 μl reaction buffer, 40 μl sample and 10 μl Ac-DEVD-pNA (2 mM) were mixed. The resulting mixture was incubated at 37°C for 16 h and the absorbance measured at 405 nm. Caspase3 activities were determined based on absorbance of produced pNA in unit sample proteins.

### Synthesis of dsRNA and knockdown of *BmDredd*

Double-stranded RNAs (dsRNA) for *BmDredd* were synthesized using a T7 RiboMAX™ Express RNAi System (Promega, Madison, WI, USA). PCR product templates were obtained using the following primers: 5’ ATGTTTCGACCTGACGCTTTA 3’ and 5’ TCATGGCTTAAATAAGTAAAG 3’. Therma cycler conditions were:98°C for 10 sec, 55°C for 15 sec, and 72°C for 50 sec for 35 cycles using Prime STAR DNA Polymerase (TaKaRa).T7 RNA polymerase promoter sequence 5’GGATCCTAATACGACTCACTATAGG3’ was added to the 5’ end of the above primers. DsRNA-*Bm*Dredd was synthesized according to the instructions. For RNA interference, each silkworm was injected with ~15 μg dsRNA-*Bm*Dredd. Control silkworm was injected with 10 μl dsRNA-EGFP. SGs were dissected 30 h post-injection for qRT-PCR and caspase3 activity detection.

### Overexpression of *BmDredd* in *Bombyx mori*

Primers were designed (Table B in S1 File). To generate the recombinant plasmid PXL-BAC-U6-Dredd-GFP, sequences for *BmU6*, *BmDredd*, and *IE-GFP-SV40* were amplified by PCR and cloned into PXL-BACII according to the protocol provided with a CloExpressMutis kit (Vazyme, Nanjing, China). The plasmid PXL-BACII was linearized using *Xba*I and *Xho*I restriction sites. The reaction system contained 4 μl 5×CE Multis Buffer, 2 μl Exnase^TM^ Multis, the above PCR products and the linearized vector. The recombinant vector (7.5 μg) was diluted into 12 μl sterile 0.4% trehalose solution and then mixed with 1 μl of TurboFect *in vivo* transfection Reagent (Thermo, Wilmington, NC, USA) immediately by pipetting. The mixture was incubated at room temperature for 20 min and injected into day 4 of the fifth larval instar silkworm. Control silkworms were injected with the same amount of 0.4% trehalose solution. After two days, the gene expression level was monitored and SGs were photographed under a fluorescence microscope (OLYMPUS BX53, Japan).

### Transmission electron microscopy

SGs were dissected from silkworms during the middle of the spinning stage and fixed in 2.5% glutaraldehyde overnight at 4°C. Specimens were rinsed with phosphate buffer solution (0.1 M, pH 7.0) three times for 15 min each time, then fixed in 1% osmium tetroxide for 1 h, rinsed again, dehydrated in an ethanol series, and then treated with mixture of eponresin and acetone (V/V = 1:1 for 1 h, V/V = 3:1 for 3 h). Samples were finally embedded in an Epon-Araldite mixture overnight. The samples were cut into slices (70 nm) and observed by transmission electron microscopy (TEM, Hitachi HT7700, Japan).

### Immunofluorescence

SGs were isolated and fixed in 4% formaldehyde overnight at 4°C. The tissues were dehydrated in an ethanol series and embedded in paraffin. Sections were sliced on a microtome (4 μm) and placed on slides. *Bm*Dredd rabbit polyclonal antibody was prepared by Huabio Ltd. Hangzhou, China. The samples were de-waxed and immersed in an ethanol series (95%, 85% and 75%) for 5 min each, washed with ddH_2_O 3 times for 5 min. Trisodium citrate repair solution was added to the sections for 4 min under high pressure. The samples were washed twice for 5 min in ddH_2_O and 3 times for 5 min with immunol staining washing buffer (Sangon) after cooling to room temperature. A 3% H_2_O_2_-methanol solution (50 μl) was added to each sample for 15 min. The samples were then rinsed three times for 5 min, blocked with 5% BSA for 45 min, incubated with *Bm*Dredd antibody (1:500) overnight at 4°C. Samples were rinsed four times for 5 min before incubation with Rhodamine-conjugated AffiniPure Goat Anti-Rabbit IgG (1:200; Huabio) without light for 1 h, followed by rinsing three time times with immunol staining washing buffer. DAPI (3.5 μg/ml) was added for 15 min at room temperature and washed three times same as above. Images were captured using a fluorescence microscope (OLYMPUS BX53) using the same light intensity.

## Results

### Comparison of expression levels of *BmDredd* with apoptosis-related genes *BmEcR* and *BmIce* in the middle and posterior silk gland during metamorphosis

**Fig 1:**
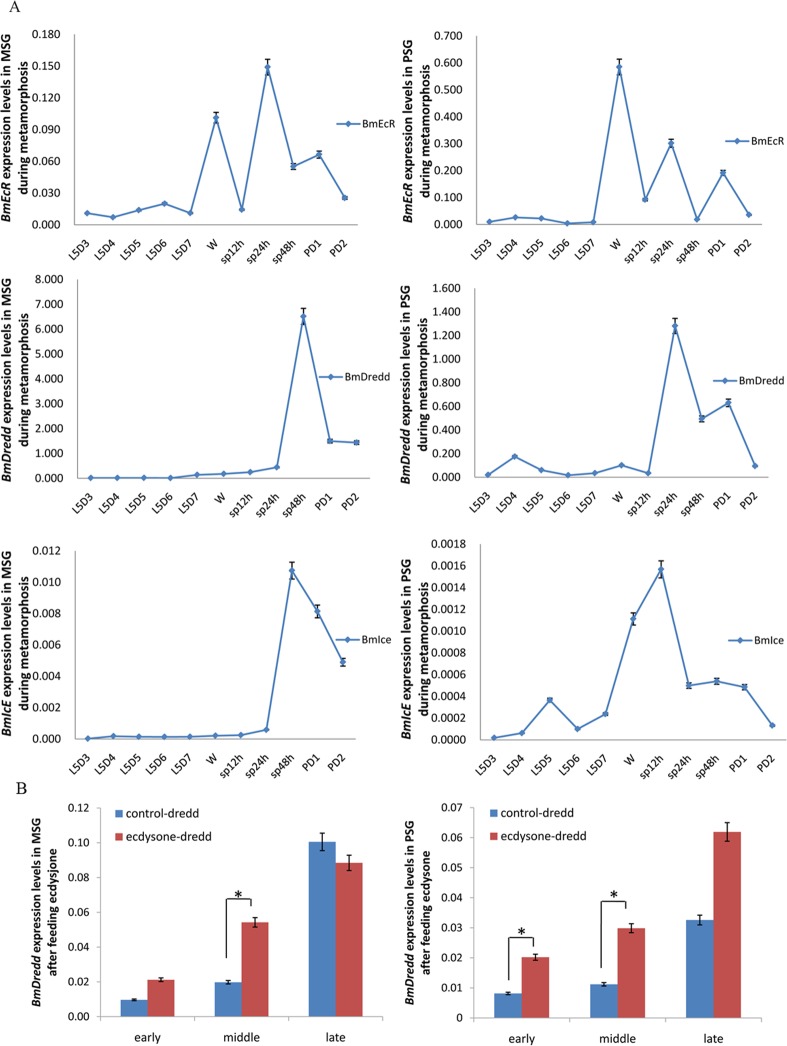
Expression level interactions between *BmDredd* and other two apoptosis-related genes in MSG and PSG during metamorphosis. (A) Expression levels in MSG and in PSG. L5D3-L5D7: day 3 to day 7 of the fifth larval instar. W: wandering stage. sp12h: 12 h of spinning. PD1: 1-day-old pupa. PD2: 2-day-old pupa. (B) The increase of *BmDredd* expression induced by ecdysone in MSG and PSG, P<0.5. Early, middle and late means the time of feeding ecdysone at L5D3, L5D5 to L5D6 and L5D7 to wandering respectively.

*Bm*EcR is the receptor of ecdysone, and *Bm*IcE is an important effector caspase. The expression level of *BmEcR* peaked twice after the wandering stage in the middle silk gland (MSG), whereas the expression levels of *BmIce* and *BmDredd* increased rapidly starting from 24 h post-spinning (sp24h) and reached the maximum expression levels at 48 h post-spinning (sp48h). The change in *BmDredd* expression was the most dramatic with an increase of over 400 times (Fig 1A). In the posterior silk gland (PSG), *BmEcR*, *BmIcE* and *BmDredd* were elevated during the larval to pupal metamorphosis stage, which is the period when SG apoptosis begins. Additionally, *BmEcR* expression was highest at the wandering stage and it peaked earlier than *BmDredd* and *BmIcE*. The expression of *BmDredd* began to rise at sp12h in the PSG and reached the highest at sp24h, whereas *BmEcR* expression began at day 7 of the fifth larval instar (L5D7) (Fig 1A). Additionally, The expression levels of *BmDredd* increased both in the MSG and PSG during early (L5D3) or middle (L5D5 to L5D6) fifth instar 24 hours after feeding ecdysone, but feeding ecdysone to later (L5D7 to wandering) fifth instar impeded the normal expression of *BmDredd* in MSG (Fig 1B).

### *BmDredd* Expression levels in different silkworm tissues and the effects of a caspase inhibitor

**Fig 2:**
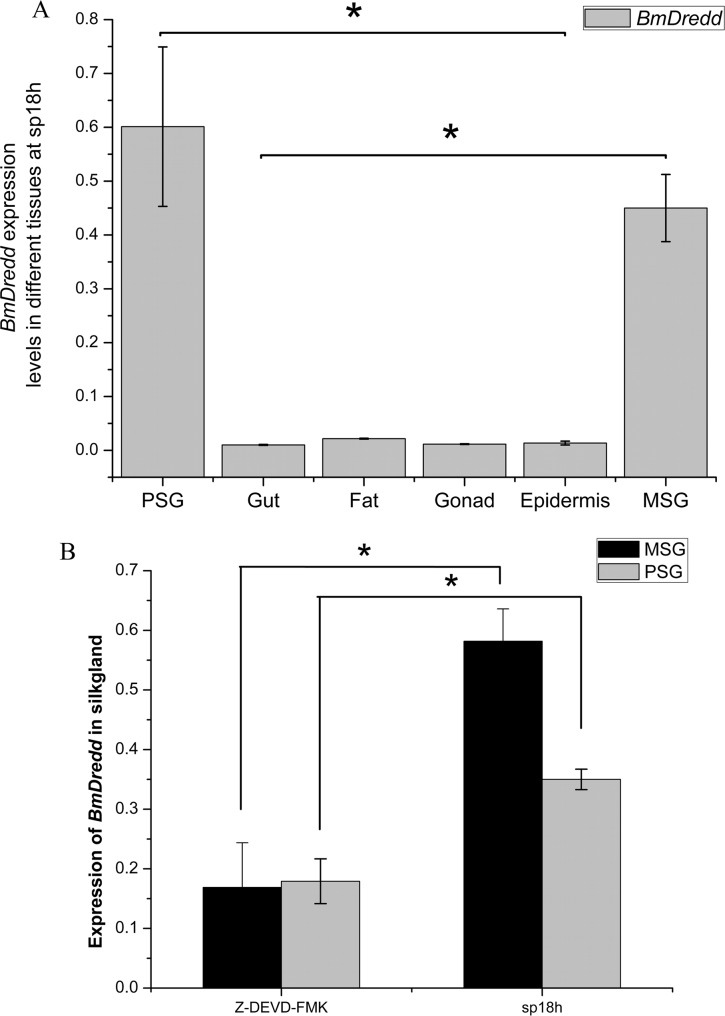
Role of *BmDredd* in SG apoptosis. (A) *BmDredd* expression levels in different silkworm tissues at sp18h, P<0.05. (B) *BmDredd* expression levels in SG after injecting Z-DEVD-fmk for about 36 h, P<0.05.

Silkworms present drastic changes during the transition from the spinning to the pupal stage. During this period, most larval tissues undergo quick degeneration forming fat. This degeneration involves apoptosis and an increase in caspase3-like activity. We found that caspase3-like activity peaked during the wandering stage in the MSG and at sp12h in the PSG (Figure C in S1 File). At 36 h after spinning, caspase3-like activity dropped to pre-wandering stage levels, but then underwent a second period of upregulation (Figure C in S1 File). In addition, we found that *BmDredd* expression in the PSG was slightly higher than the MSG (Fig 2A), but expression in both tissues was significantly higher (P<0.05) than the other five tissues (fat, gut, gonad and epidermis) (Fig 2A). Moreover, addition of the caspase inhibitor Z-DEVD-fmk to silkworm significantly decreased the expression of *BmDredd* by ~71% and 49% in the MSG and PSG respectively (Fig 2B).

### RNAi of *BmDredd* delayed the SG degeneration process

SG nuclei are initially rod-shaped in first instar larvae, but then begin to gradually branch out in the later instar stages. By the fifth instar, SG nuclei are highly branched. Nuclei in these stages had both condensed and dispersed chromatin. Typically, the more dispersed chromatin, the higher the DNA synthesis activity. We found that the amount of dispersed chromatin was significantly reduced in late fifth instars.

**Fig 3:**
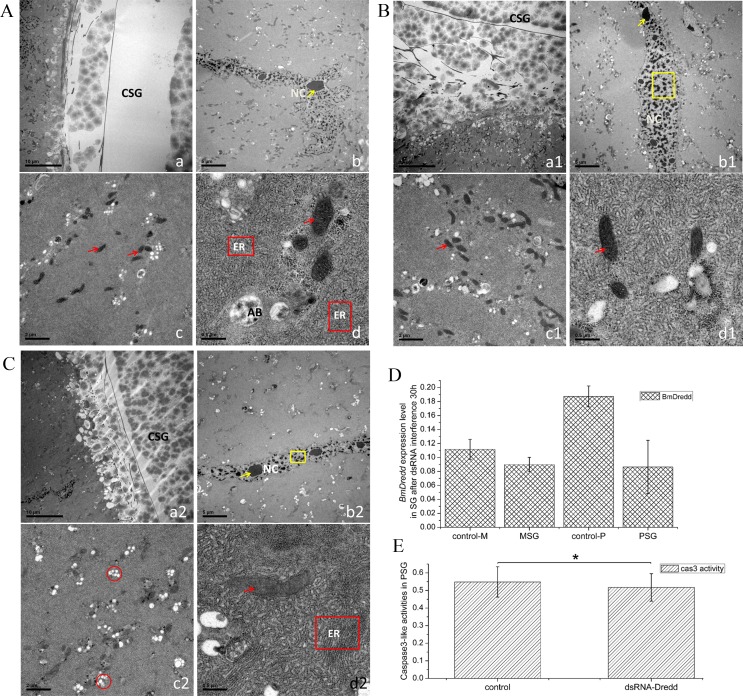
TEM of the SG during the middle of the spinning stage. (a), (a1) and (a2): cavity of SG (CSG). (b), (b1) and (b2):nucleus of SG. (c to c2) and (d to d2): cytoplasm. Yellow arrows in b, b1 and b2: condensed chromatin, yellow frames in b1 and b2: dispersed chromatin. Red arrows in c, c1, and d to d2: mitochondrion, red frames in d and d2: endoplasmic reticulum (ER), red circles in c2: golgi apparatus. AB: apoptosis body. A: Control SG injecting with dsRNA-EGFP at L5D7. B: dsRNA injected during the middle of the fifth instar (about middle of L5D6). C: dsRNA injected during late stage of the fifth instar (about early wandering). D: *BmDredd* expression levels in SG at 30 h post-*BmDredd* interference. E: Caspase3-like activities in PSG after dsRNA treatments for about 30 h, P<0.5.

RNAi-mediated knockdown decreased the *BmDredd* expression in the SG (Fig 3D), in particular in the PSG (~54% reduction). The TEM samples were taken from PSG during the middle of the spinning stage. We were able to identify fibroin proteins in Fig 3A which had completed secretion. As a result, the SG cavity was empty (Fig 3A:a). In addition, condensed chromatin increased and the nucleus (NC) was disordered (Fig 3A:b). Mitochondria (Fig 3A:c,d: red arrow) decreased in size, the structure of the ER was almost invisible, and apoptosis bodies appeared. In Fig 3B and 3C, we could see fibroin protein secretion and there was less condensed chromatin in Fig 3B than in Fig 3A and 3C. The ER was more developed in Fig 3B and 3C. More Golgi apparatus (Fig 3B:c2:red circles) and normal-functioning mitochondria were visible in Fig 3C. The caspase3 activity also showed a decrease after dsRNA interference (Fig 3E). Thus the dsRNA interference inhibited the apoptosis of the SG.

### Transient expression of *BmDredd* caused apoptosis of silk gland

**Fig 4:**
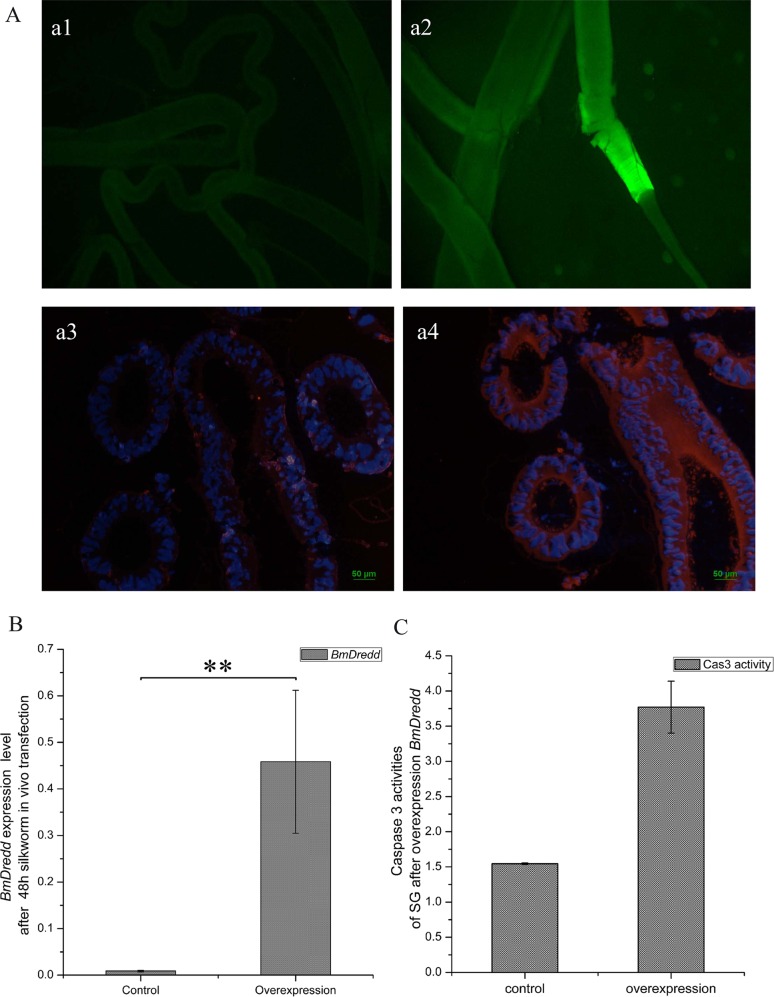
Overexpression of *BmDredd* in silkworm. (A) Fluorescence microscopy, a1 and a2: green fluorescence indicative of successful transfection and overexpression of the PXL-BAC-U6-Dredd-GFP. a3 and a4: DAPI and Rhodamine staining, DAPI (blue fluorescence): nuclei, Rhodamine (red fluorescence): *Bm*Dredd. a1, a3: control, a2, a4: overexpression of *Bm*Dredd. (B) *BmDredd* expression levels in the SG after 48h overexpression. P<0.01. (C) caspase3-like activity of SG after 48 h overexpression.

Green fluorescence was detected in SG 48 h-post transient transfection (Fig 4A:a2), whereas no fluorescence was detected in controls (Fig 4A:a1). Results of immunofluorescence showed that *Bm*Dredd red flourescence was predominantly located in the cytoplasm of the SG interior cavity wall (Fig 4A:a4) and protein levels increased after transfection with the overexpression plasmid (Fig 4A:a3, a4). *BmDredd* transcription levels increased by ~51 times over control levels (Fig 4B) and caspase3-like activity increased more than two-fold (Fig 4C).

## Discussion

Prior studies have shown that *BmEcR*, *BmDredd* and *BmIce* are important apoptosis-related genes [17]. We investigated their expression profile in SG and found that *BmDredd*, *BmEcR* and *BmIce* transcription levels significantly increased in the MSG and PSG during the larval-pupal transition (Fig 1A). Expression of *BmEcR*, the receptor for ecdysone, changes with ecdysone levels [15]. Recent studies have demonstrated that *Bm*EcR responds to molting hormone (ecdysone) to active a genetic regulated cascade that results in tissue degeneration during metamorphosis. We found that the elevation in *BmDredd* expression closely followed (~24 h) *BmEcR* expression (Fig 1A), and was significantly increased compare to controls after feeding ecdysone in early or middle fifth instar larvae (Fig 1B). These findings indicate that *BmDredd* functions in SG degeneration and it is most likely a downstream gene of *BmEcR*. Since *BmDredd* belongs to the silkworm caspase family, similar to the effector caspase *BmIce-2*, it is likely an initiator caspase homolog. We suggest that *BmDredd* can induce apoptosis with similar functions as *BmIce-2*. Furthermore, the expression levels of *BmDredd* were significantly elevated in SG compared to other tissues (Fig 2A) demonstrating that *BmDredd *played a remarkable role in SG apoptosis.

Caspase3 activity is a vital index for detecting apoptosis. The results indicated that apoptosis occurred in MSG and PSG from L5D7 through the spinning stage to pupa, with the apoptosis rate in MSG slightly more advanced than PSG and peaked at the wandering stage (Figure C in S1 File). This activity profile is reminiscent of *BmDredd* expression at sp12h (Fig 2A). Z-DEVD-fmk is a specific, irreversible caspase3 inhibitor, which also potently inhibits caspase6, caspase7, caspase8 and caspase10. Z-DEVD-fmk also inhibited caspase3-like activities in SG (Figure D in S1 File) and the expression of *BmDredd* (Fig 2B). Therefore, we speculate that SG apoptosis is associated with *BmDredd* expression. In *Drosophila*, caspase competence is continuous from early in day 3 larvae, but it cannot induce apoptotic execution because of high DIAP1 levels block caspase activation [6]. While the specific regulatory mechanism used in silkworms is currently unknown, our results suggest that control of caspase competence in SG apoptosis is likely similar to that used in *Drosophila*.

dsRNA-*Bm*Dredd reduced *BmDredd* expression in the SG (Fig 3D). After dsRNA treatment, the SG cavity retained fibroin secretion with more dispersed nuclei than controls (Fig 3B:b1). Normal mitochondria and endoplasmic reticulum structures (Fig 3B:d2) were found in *BmDredd* knockdown SG cells. There were no or few apoptosis bodies in Fig 3B and 3C, however, they were abundant in the control (Fig 3A:d). These changes in microstructures illustrated that knockdown of *BmDredd* delayed SG apoptosis. However, mitochondria and condensed chromatin were also found in Fig 3B and 3C respectively. dsRNA treatment during the middle of the fifth instar hindered nucleus condensation. Given that RNAi effects for a short period of time, the condensing of the cytoplasm was not prevented. During late fifth instars, apoptosis of nuclei has already occurred before adding dsRNA-*Bm*Dredd, hence a certain degree of condensed nuclei were visible. Based on these findings, we inferred that apoptosis of SG may initiate from the nucleus and *BmDredd* may be an important gene to induce nuclear condensation. This result was consistent with the study of Masatoshi Iga in 2007 [7].

*In vivo* transfection reagent is ideal for nucleic acid delivery [28, 29]. The protocol is easy and versatile that it has been adapted to many other species including mice, rat, chicken and mosquito [30–33]. We transiently overexpressed the *BmDredd* in vivo in silkworm SG using TurboFect *in vivo* transfection Reagent (Fig 4A and 4B). The overexpression leads to light apoptosis of the SG (Fig 4C). However, whether stable overexpression of *BmDredd* could induce extensive apoptosis of the SG remains unknown.

In conclusion, *BmDredd* regulated SG apoptosis and is probably a downstream gene of *BmEcR*. Knockdown of *BmDredd* impeded SG apoptosis and overexpressing *BmDredd* triggered SG apoptosis. More research, however, is required to fully reveal the molecular mechanisms underlying SG apoptosis.

## Supporting Information

S1 File **Fig A. Different stage of the silk gland of the silkworm during the fifth larval.** A-F: L5D3-L5D8. Bar:1 cm. The SGs were dissected from Qiu feng × Bai Yu strain.
**Fig B. The transformation of silk gland of silkworm during the larval to pupal.** (A) the wandering stage(W). (B) middle of the spinning day. (C) prepupal stage(PP). (D) the second day of pupal. Bar:1 cm. These were dissected from Qiu feng x Bai Yu strain.
**Fig C. Caspase3-like activities of SG from L5D4 to PD1.** (A) Caspase3-like activities in MSG. (B) Caspase3-like activities in PSG.
**Fig D. Caspase3-like activities of MSG and PSG after Z-DEVD-fmk treatment**.
**Table A. Primers used in real-time PCR**.
**Table B. Primers used for vector construction**.
(DOCX)

## References

[1] Wyllie AH, Kerr JF, Currie AR. Cell death: the significance of apoptosis. Int Rev Cytol. 1980; 68:251–306. 7014501 10.1016/s0074-7696(08)62312-8

[2] Thornberry NA, Lazebnik Y. Caspases: Enemies Within. Science 1998; 281: 1312–1316. 9721091 10.1126/science.281.5381.1312

[3] Reed JC. Mechanisms of apoptosis. American Journal of Pathology 2000; 157:1415–1430. 10.1016/S0002-9440(10)64779-7 11073801 PMC1885741

[4] Li QR, Deng XJ, Yang WY, Huang ZJ, Tettamanti G, Cao Y, et al Autophagy, apoptosis, and ecdysis-related gene expression in the silk gland of the silkworm (*Bombyx mori*) during metamorphosis. Canadian Journal of Zoology 2010; 88:1169–1178.

[5] Kakei M, Iwami M, Sakurai S. Death commitment in the anterior silk gland of the silkworm, *Bombyx mori*. J Insect Physiol. 2005; 51:17–25. 10.1016/j.jinsphys.2004.10.012 15686642

[6] Yin VP, Thummel CS, Bashirullah A. Down-regulation of inhibitor of apoptosis levels provides competence for steroid-triggered cell death. Journal of Cell Biology 2007; 178:85–92. 10.1083/jcb.200703206 17591924 PMC2064425

[7] Iga M, Iwami M, Sakurai S. Nongenomic action of an insect steroid hormone in steroid-induced programmed cell death. Molecular & Cellular Endocrinology 2007; 263:18–28. 17045392 10.1016/j.mce.2006.08.005

[8] Nagaraju J, Goldsmith MR. Silkworm genomics-Progress and prospects. Current Science 2001; 83(4):415–425.

[9] Zhang CD, Li FF, Chen XY, Huang MH, Zhang J, Cui HJ, et al DNA replication events during larval silk gland development in the silkworm, *Bombyx mori*. J Insect Physiol. 2012; 58:974–978. 10.1016/j.jinsphys.2012.04.017 22609363

[10] Ji MM, Liu AQ, Gan LP, Xing R, Wang H, Sima YH, et al Functional analysis of 30K proteins during silk gland degeneration by a caspase-dependent pathway in *Bombyx mori*. Insect Mol Biol. 2013; 22:273–283. 10.1111/imb.12019 23496335

[11] Dubrovsky EB. Hormonal cross talk in insect development. Trends in Endocrinology & Metabolism 2005; 16:6–11. 15620543 10.1016/j.tem.2004.11.003

[12] Matsui H, Kakei M, Iwami M, Sakurai S. Hormonal regulation of the death commitment in programmed cell death of the silkworm anterior silk glands. J Insect Physiol. 2012; 58:1575–1581. 10.1016/j.jinsphys.2012.09.012 23063728

[13] Yin VP, Thummel CS. Mechanisms of steroid-triggered programmed cell death in *Drosophila*. Seminars in Cell & Developmental Biology 2005; 16:237–243. 15797834 10.1016/j.semcdb.2004.12.007

[14] Terashima J, Yasuhara N, Iwami M, Sakurai Sho. Programmed cell death triggered by insect steroid hormone, 20-hydroxyecdysone, in the anterior silk gland of the silkworm, *Bombyx mori*. Dev Genes Evol. 2000; 210:545–558. 10.1007/s004270050345 11180805

[15] Tsuzuki S, Iwami M, Sakurai S. Ecdysteroid-inducible genes in the programmed cell death during insect metamorphosis. Insect Biochem. Mol. Biol. 2001;31:321–331. 11222941 10.1016/s0965-1748(00)00124-7

[16] Jia SH, Li MW, Zhou B, Liu WB, Zhang Y, Miao XX, et al Proteomic Analysis of Silk Gland Programmed Cell Death during Metamorphosis of the Silkworm *Bombyx mori*. J Proteome Res. 2007; 6:3003–3010. 10.1021/pr070043f 17608510

[17] Zhang JY, Pan MH, Sun ZY, Huang SJ, Yu ZS, Liu D, et al The genomic underpinnings of apoptosis in the silkworm, *Bombyx mori*. BMC Genomics 2010; 11:611 10.1186/1471-2164-11-611 21040523 PMC3091752

[18] Czerski L, Nunez G. Apoptosome formation and caspase activation: is it different in the heart? J Mol Cell Cardiol. 2004; 37:643–652. 10.1016/j.yjmcc.2004.04.016 15350837

[19] Xie K, Tian L, Guo XY, Li K, Li JP, Deng XJ, et al *Bm*ATG5 and *Bm*ATG6 mediate apoptosis following autophagy induced by 20-hydroxyecdysone or starvation. Autophagy 2016; 12:381–396. 10.1080/15548627.2015.1134079 26727186 PMC4836027

[20] Yi HS, Pan CX, Pan C, Song J, Hu YF, Wang L, et al *Bm*ICE-2 is a novel pro-apoptotic caspase involved in apoptosis in the silkworm, *Bombyx mori*. Biochem Bioph Res Co. 2014; 445:100–106. 10.1016/j.bbrc.2014.01.139 24491540

[21] Zhang JY, Xu W, Pan C, Yi HS, Hu YF, Song J, et al Cloning and Characterization of the Caspase Family Member *Bm*Dronc from *Bombyx mori* (Lepidoptera: Bombycidae) Embryo Cells. Entomological Society of America 2013; 106:265–272.

[22] Huang QH, Deveraux QL, Maeda S, Stennick HR, Hammock BD, Reed JC. Cloning and characterization of an inhibitor of apoptosis protein (IAP) from *Bombyx mori*. Biochim. Biophys. Acta. 2001; 1499:191–198. 11341966 10.1016/s0167-4889(00)00105-1

[23] Pan C, Hu YF, Yi HS, Song J, Wang L, Pan MH, et al Role of *Bm*buffy in hydroxycamptothecine-induced apoptosis in *Bm*N-SWU1 cells of the silkworm, *Bombyx mori*. Biochem Bioph Res Co. 2014; 447:237–243. 10.1016/j.bbrc.2014.03.093 24690173

[24] Jin KY. Death effector domain for the assembly of death-inducing signaling complex. Apoptosis 2015; 20:235–239. 10.1007/s10495-014-1060-6 25451007

[25] Chen RT, Jiao P, Lu Y, Xin HH, Zhang DP, Wang MX, et al *Bm*Dredd regulates the apoptosis coordinating with *Bm*Daxx, *Bm*cide-b, *Bm*Fadd, and *Bm*Creb in *Bm*N cells. Arch Insect Biochem Physiol. 2016; 10.1002/arch.21349 27558456

[26] Wang L, Song J, Bao XY, Chen P, Yi HS, Pan HM, et al *Bm*Dredd is an initiator caspase and participates in Emodin-induced apoptosis in the silkworm, *Bombyx mori*. Gene 2016; 591:362–368. 10.1016/j.gene.2016.06.018 27291821

[27] Livak KJ, Schmittgen TD. Analysis of relative gene expression data using real-time quantitative PCR and the 2(-Delta Delta C(T)) Method. Methods 2001; 25:402–408. 10.1006/meth.2001.1262 11846609

[28] Linemann T, Thomsen LB, Jardin KG, Laursen JC, Jensen JB, Lichota J, et al Development of a novel lipophilic, magnetic nanoparticle for in vivo drug delivery. Pharmaceutics 2013; 5(2):246–260. 10.3390/pharmaceutics5020246 24300449 PMC3834948

[29] Mohammadi Z, Dorkoosh FA, Hosseinkhani S, Gilani K, Amini T, Najafabadi AR, et al In vivo transfection study of chitosan-DNA-FAP-B nanoparticles as a new non viral vector for gene delivery to the lung. Int J Pharm. 2011; 421(1):183–188. 10.1016/j.ijpharm.2011.09.029 21979252

[30] Bhang HE, Gabrielson KL, Laterra J, Fisher PB, Pomper MG. Tumor-specific imaging through progression elevated gene-3 promoter-driven gene expression. Nat Med. 2011; 17(1):123–129. 10.1038/nm.2269 21151140 PMC3057477

[31] McKnight CD, Winn SR, Gong X, Hansen JE, Wax MK. Revascularization of rat fasciocutaneous flap using CROSSEAL with VEGF protein or plasmid DNA expressing VEGF. Otolaryngol Head Neck Surg. 2008; 139(2):245–249. 10.1016/j.otohns.2008.04.014 18656723

[32] Jordan BJ, Vogel S, Stark MR, Beckstead RB. (2014) Expression of green fluorescent protein in the chicken using in vivo transfection of the piggyBac transposon. J Biotechnol. 2014; 173:86–89. 10.1016/j.jbiotec.2014.01.016 24452099

[33] Peng R, Maklokova VI, Chandrashekhar JH, Lan Q. In vivo functional genomic studies of sterol carrier protein-2 gene in the yellow Fever mosquito. PLoS One 2011; 6(3): e18030 10.1371/journal.pone.0018030 21437205 PMC3060925

