# Supplementary material for: Role of BmDredd during Apoptosis of Silk Gland in Silkworm, Bombyx mori
Source: PLoS One. 2017 Jan 9;12(1):e0169404. doi: 10.1371/journal.pone.0169404 (PMC5222620; doi:10.1371/journal.pone.0169404)
Supplement: S1 File — Fig A. Different stage of the silk gland of the silkworm during the fifth larval. A-F: L5D3-L5D8. Bar:1 cm. The SGs were dissected from Qiu feng × Bai Yu strain. Fig B. The transformation of silk gland of silkworm during the larval to pupal. (A) the wandering stage(W). (B) middle of the spinning day. (C) prepupal stage(PP). (D) the second day of pupal. Bar:1 cm. These were dissected from Qiu feng x Bai Yu strain. Fig C. Caspase3-like activities of SG from L5D4 to PD1. (A) Caspase3-like activities in MSG. (B) Caspase3-like activities in PSG. Fig D. Caspase3-like activities of MSG and PSG after Z-DEVD-fmk treatment. Table A. Primers used in real-time PCR. Table B. Primers used for vector construction. (DOCX) [file pone.0169404.s001.docx]

**Table A. Primers used in real-time PCR**

| Purposes | Gene names | Sequences (5’-3’) |
| --- | --- | --- |
| Real-time PCR primers | *BmDredd* | F: TACTGGGCAACAGCACCT |
|  |  | R: ATGGGAACCTGAGGATGA |
|  | *BmEcR* | F: TCTTCGGCACTGGGTTTG |
|  |  | R: TTGTGGGAGGCATTGGTA |
|  | *BmIce* | F: GCCGACCAACCATACAAG |
|  |  | R: TCTGCCCTCATCCATAAA |
| Real-time PCR primers for the normalization | *BmActin3* | F: GCGCGGCTACTCGTTCACTACC |
|  |  | R: GGATGTCCACGTCGCACTTCA |

**Table B. Primers used for vector construction**

| Genes | Sequences (5’ -3’) |
| --- | --- |
| *BmDredd* | F: ATGTTTCGACCTGACGCTTTA |
|  | R: CCCGAACTGCAAATCCTATCATGGCTTAAATAAGTAAAG |
| *BmU6* | F: GGGGGATCCACTAGTTCTAGAAGGTTATGTAGTACACATTG |
|  | R: GTCAGGTCGAAACATACTTGTAGAGCACGATATT |
| *IE-GFP-SV40* | F: GATTTGCAGTTCGGGACATAAATG |
|  | R: ATCGATACCGTCGACCTCGAGCCAAACTGGAACAACACTCAACC |


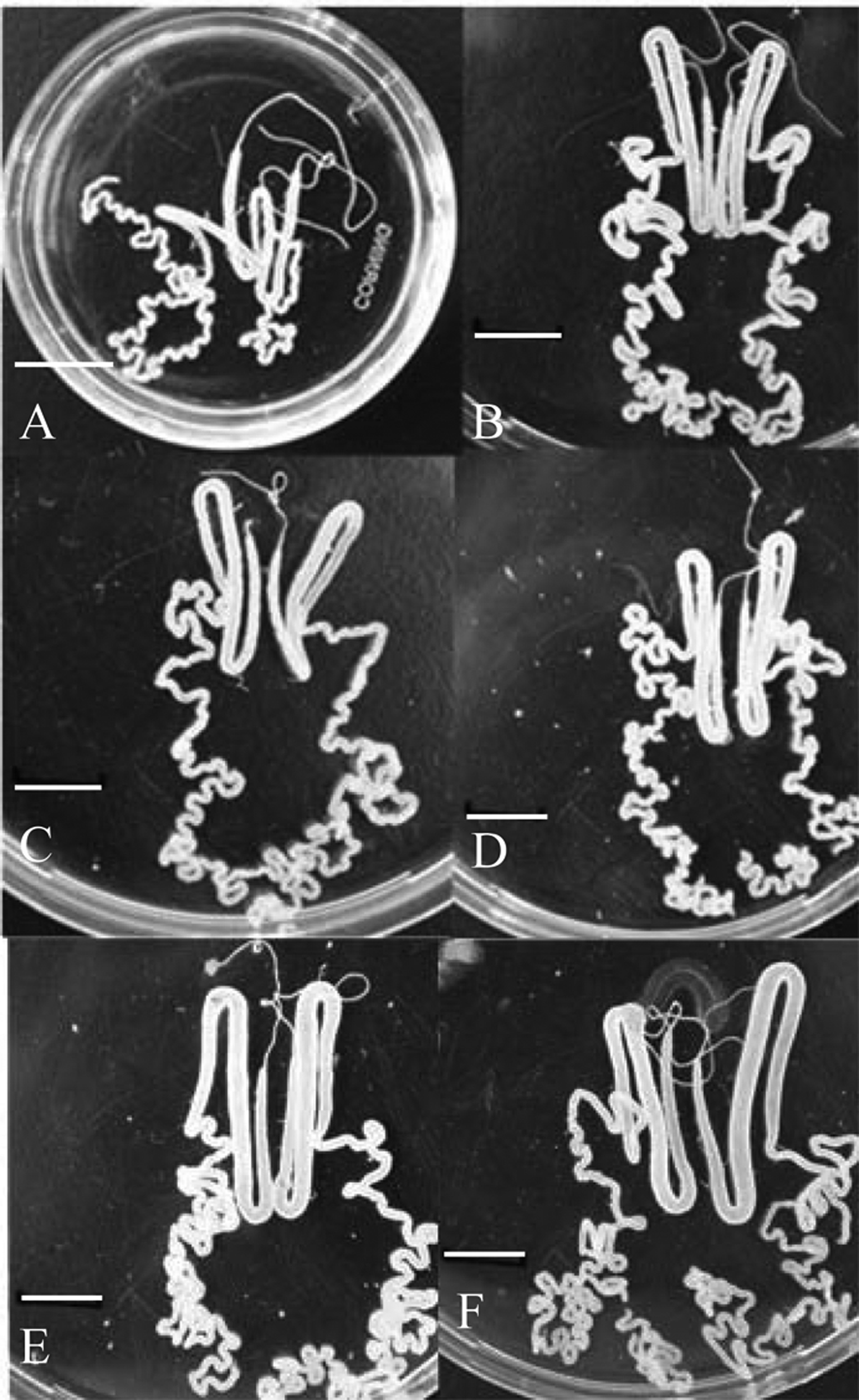


**Figure A.** **Different stage of the silk gland of the silkworm during the fifth larval.** A-F: L5D3-L5D8. Bar:1cm. The SGs were dissected from Qiu feng x Bai Yu strain.


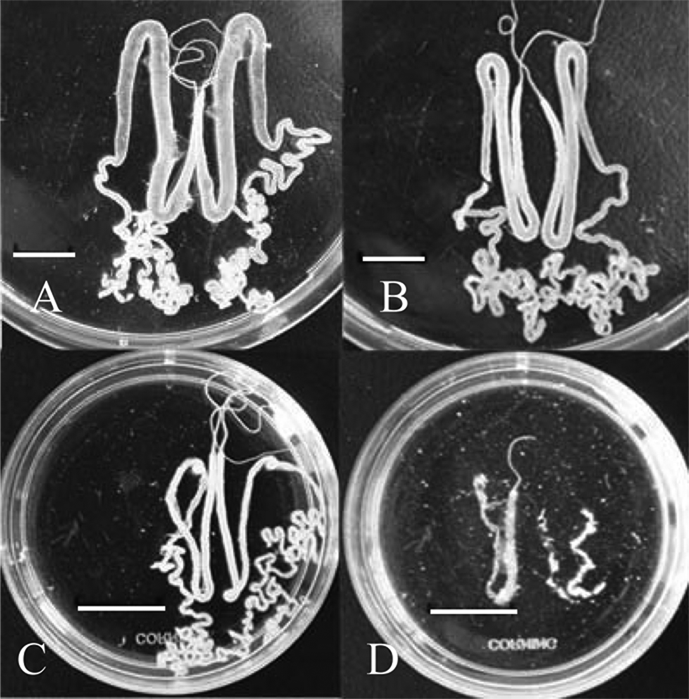


**Figure B. The transformation of silk gland of silkworm during the larval to pupal.** (A) the wandering stage(W). (B) middle of the spinning day. (C) prepupal stage(PP). (D) the second day of pupal. Bar:1cm. These were dissected from Qiu feng x Bai Yu strain.


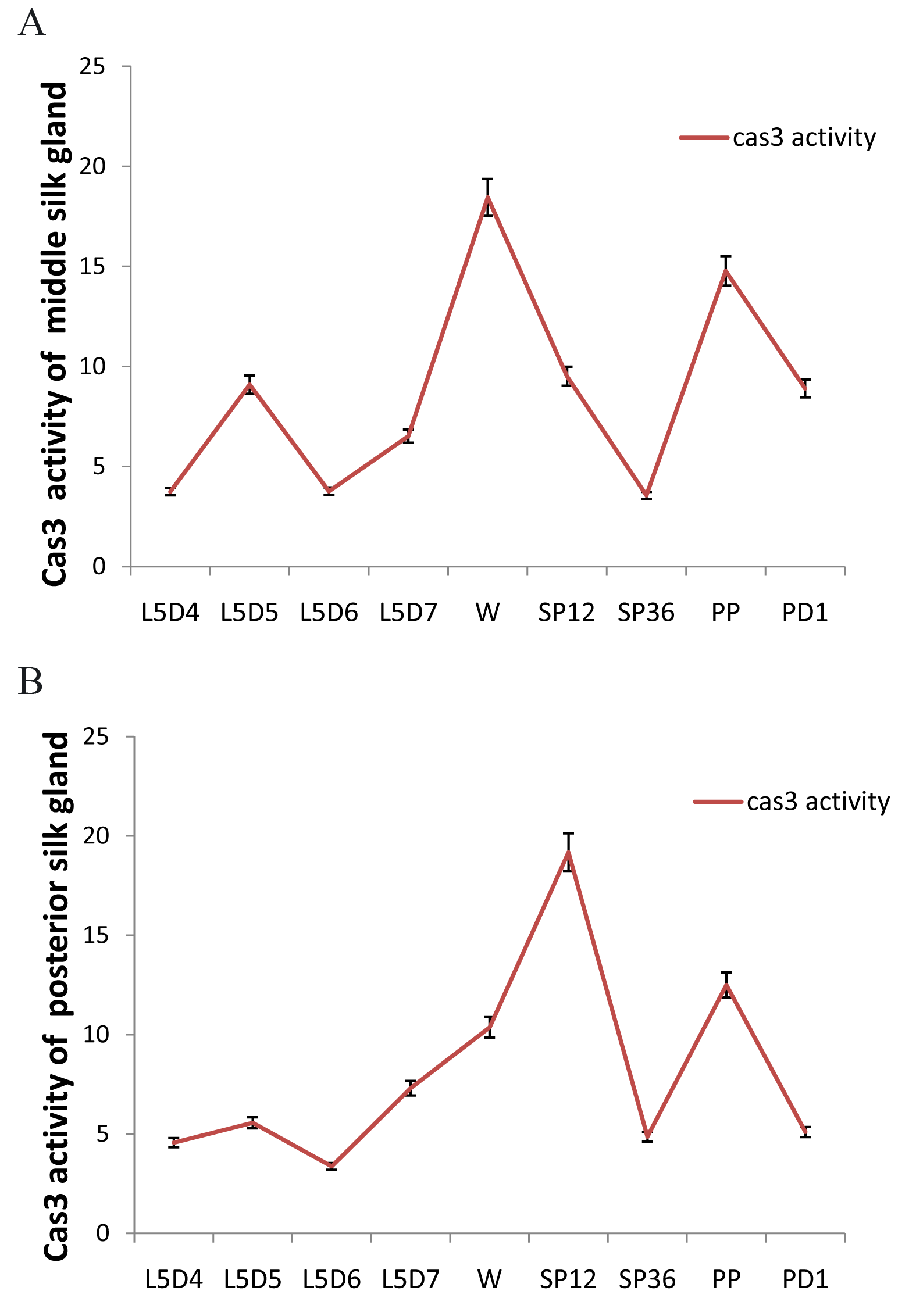


**Figure C. Caspase3-like activities of SG from L5D4 to PD1.** (A) Caspase3-like activities in MSG. (B) Caspase3-like activities in PSG.


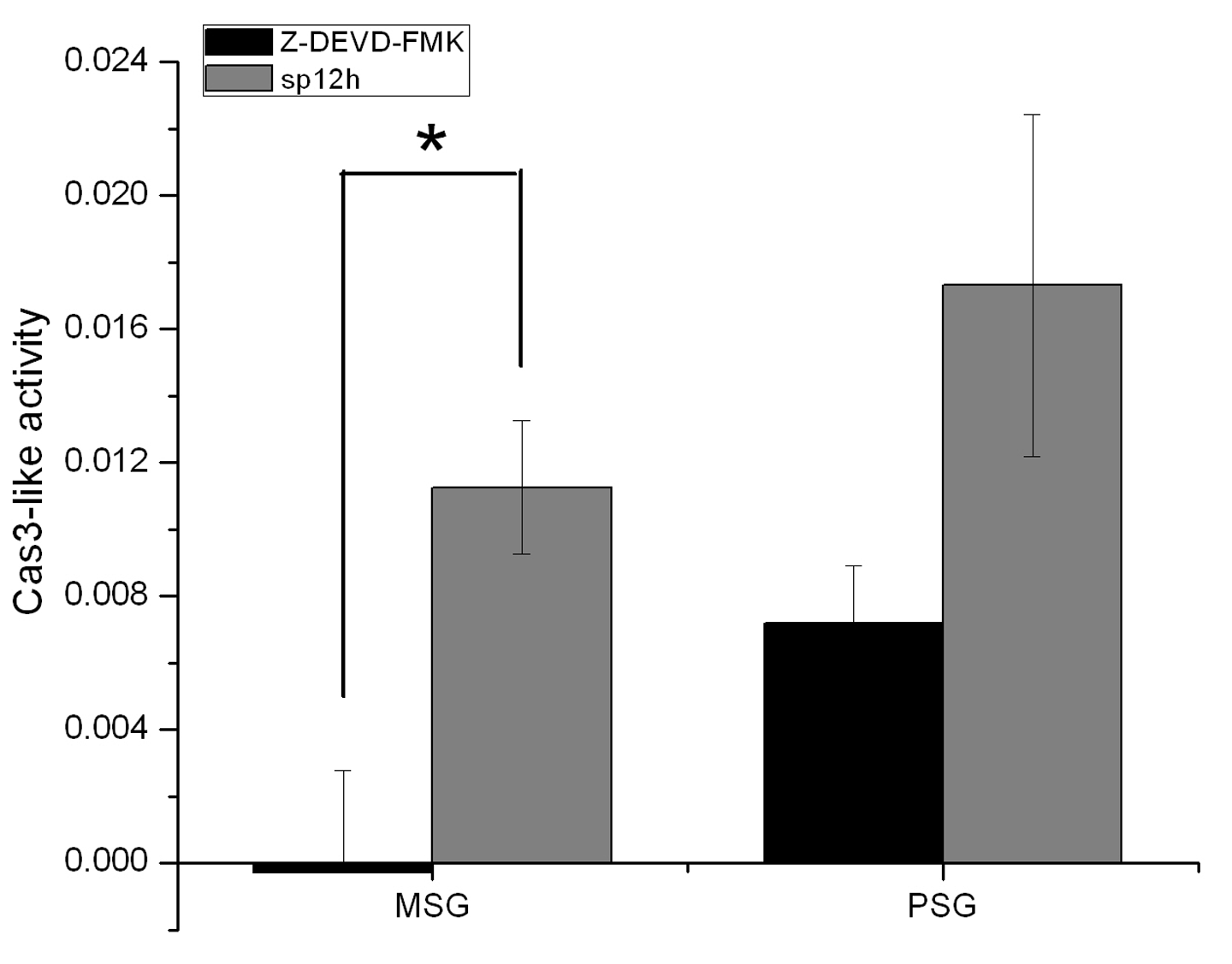


**Figure D. Caspase3-like activities of MSG and PSG after Z-DEVD-fmk treatment.**
